# Two kinds of memory signals in neurons of the human hippocampus

**DOI:** 10.1073/pnas.2115128119

**Published:** 2022-05-05

**Authors:** Zhisen J. Urgolites, John T. Wixted, Stephen D. Goldinger, Megan H. Papesh, David M. Treiman, Larry R. Squire, Peter N. Steinmetz

**Affiliations:** ^a^Department of Psychology, University of California San Diego, La Jolla, CA 92093;; ^b^Department of Psychology, Arizona State University, Tempe, AZ 85287;; ^c^Department of Psychology, New Mexico State University, Las Cruces, NM 88003;; ^d^Department of Neurology, Barrow Neurological Institute, Phoenix, AZ 85013;; ^e^Veterans Affairs Medical Center, San Diego, CA 92161;; ^f^Department of Psychiatry, University of California San Diego, La Jolla, CA 92093;; ^g^Department of Neurosciences, University of California San Diego, La Jolla, CA 92093;; ^h^Department of Psychology, University of California San Diego, La Jolla, CA 92093;; ^i^Neurtex Brain Research Institute, Dallas, TX 75225

**Keywords:** human hippocampus, single-unit activity, sparsely coded memory, episodic memory

## Abstract

Prior studies of the neural representation of episodic memory in the human hippocampus have identified generic memory signals representing the categorical status of test items (novel vs. repeated), whereas other studies have identified item specific memory signals representing individual test items. Here, we report that both kinds of memory signals can be detected in hippocampal neurons in the same experiment. We recorded single-unit activity from four brain regions (hippocampus, amygdala, anterior cingulate, and prefrontal cortex) of epilepsy patients as they completed a continuous recognition task. The generic signal was found in all four brain regions, whereas the item-specific memory signal was detected only in the hippocampus and reflected sparse coding. That is, for the item-specific signal, each hippocampal neuron responded strongly to a small fraction of repeated words, and each repeated word elicited strong responding in a small fraction of neurons. The neural code was sparse, pattern-separated, and limited to the hippocampus, consistent with longstanding computational models. We suggest that the item-specific episodic memory signal in the hippocampus is fundamental, whereas the more widespread generic memory signal is derivative and is likely used by different areas of the brain to perform memory-related functions that do not require item-specific information.

## Significance

Episodic memories represent the “what,” “when,” and “where” of specific episodes. In epilepsy patients, we detected single-unit activity reflecting episodic memory only in the hippocampus. This neural signal is sparsely coded and pattern-separated, consistent with predictions from neurocomputational models. We also detected single-unit activity reflecting a generic memory signal, coding whether an item is old or new without item-specific episodic information. Similar to concept cells, this generic repetition/novelty neural signal was found in multiple brain regions, including the hippocampus. In contrast, the item-specific signal was found only in the hippocampus. Our results indicate the coexistence of two memory signals in the human brain and suggest that the sparsely coded, hippocampus-specific signal is fundamental, whereas the often-studied generic signal is derivative.

The hippocampus is essential for the formation of declarative (conscious) memory (1, 2), including both episodic memory (memory for events) and semantic memory (factual knowledge). Episodic memories represent the “what, when, and where” information about remembered events (3). Here, we focus on the neural representation of episodic memory for events, specifically words presented and later repeated in a continuous recognition memory format (4).

Bilateral hippocampal lesions result in substantial anterograde amnesia for new events, whether memory is tested by recall or recognition (5). By contrast, bilateral lesions to a more anterior medial temporal lobe structure―the amygdala―have no such effect (6). One might therefore expect to find single-unit activity associated with episodic memory in the hippocampus but not in the amygdala. Yet, the earliest single-neuron studies failed to detect hippocampal neurons that fired differentially to recently presented test items vs. novel items. This was true in studies with humans (7, 8) and monkeys (9–11). One early study with monkeys identified a few such neurons in the hippocampus (12), and other studies found them in areas other than the hippocampus (e.g., inferomedial temporal cortex or inferotemporal temporal cortex) (9–11, 13, 14). Overall, this was not the pattern anticipated from lesion studies.

Subsequent studies successfully detected some memory-related neural activity (15–17), observing that ∼10% of hippocampal neurons exhibited differential firing rates based on item status, with some firing more for repeated items and others firing more for novel items. Surprisingly, similar “memory-selective” neurons were also reliably detected in the amygdala at approximately the same frequency. Yet, these memory-selective neurons responded differentially to the generic, categorical status of test items (repeated vs. novel) and thus are not episodic memory signals (i.e., signals representing memory for specific events). According to neurocomputational models dating back to Marr (18), episodic memory representations in the hippocampus are supported by sparse neural codes (19–21). If memories for individual items are sparsely coded in largely nonoverlapping (pattern-separated) neural assemblies, it should be possible to find neurons that respond to particular repeated items, rather than to an item’s generic status. Two recent single-unit studies with humans detected such neurons in the hippocampus, but not in the amygdala (22, 23), apparently reflecting sparsely coded episodic memories. In the present study, we tested 1) whether the generic and the item-specific signals coexist in neural firing patterns recorded during the same memory task, and 2) whether the two kinds of signals are present exclusively in the hippocampus or are also evident in other brain regions.

During a continuous recognition memory procedure, neurons were simultaneously recorded from four brain regions: hippocampus, amygdala, anterior cingulate cortex, and prefrontal cortex. Altogether, 55 continuous recognition memory sessions were completed by 34 epilepsy patients who had implanted clinical depth electrodes with microwires measuring single-unit activity (SUA) and multiunit activity bilaterally (24). We limited the present analyses to SUA. Words were presented consecutively and repeated once after varying lags; patients judged each word as either “novel” or “repeated.” Thus, repeated words differed from their earlier presentations as novel words only with respect to their combined “what, when, and where” episodic status (3).

## Results

### Behavioral Performance.

Average behavioral performance was well above chance and below ceiling: 83.5 ± 2.0% correct rejections for first word presentations, and 80.6 ± 2.8% hits for repeated words. The average *d*′ across sessions was 2.20 (min = 0.12, max = 3.69).

### Identifying the Generic Memory Signal.

**Fig. 1.**
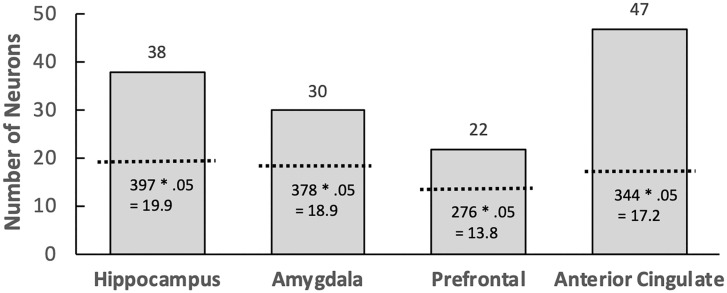
Number of neurons that exhibited generic memory signals (repeated vs. novel) in the hippocampus, amygdala, prefrontal cortex, and anterior cingulate. In each of the four regions, the total number of neurons exhibiting a generic memory signal exceeded the number expected by chance, using an alpha level of 0.05 (the dotted line) based on the total number of neurons recorded per region. For example, 38 neurons in the hippocampus exhibited a generic memory signal, which was significantly above the 19.9 expected by chance.

A generic memory signal would be evident if neurons produced firing rates that differentiate novel and repeated items. In prior work, some memory-selective neurons exhibited higher firing rates for repeated items (repetition detectors),* whereas others exhibited higher firing rates for novel items (novelty detectors). This pattern was also observed in our data using the same method that was used in prior work (13–15). Aggregated across sessions and patients, 38 hippocampal neurons (out of 397 recorded; 9.6%) exhibited statistically significant repetition/novelty signals (Fig. 1, see *SI Appendix* for representative raster plots). Of these memory-selective neurons, 20 were repetition detectors and 18 were novelty detectors (see *Materials and Methods*). Using an alpha level of 0.05, the expected number of memory-selective neurons detected by chance would be 397 × 0.05 = 19.9. The observed number of 38 was greater than chance (*P* < 0.001, Fig. 1). These memory-selective neurons were active across entire sessions, changing their firing rates in response to either repeated or novel items enough to generate statistically reliable effects.

We found a similar pattern in the amygdala, where 30 neurons (out of 378, or 7.9%) exhibited significant repetition/novelty signals (Fig. 1). There were 10 repetition detectors and 20 novelty detectors. The expected number of neurons detected by chance would be 378 × 0.05 = 18.9. The observed number of 30 was greater than chance (*P* < 0.01). We repeated this analysis in areas beyond the medial temporal lobe (i.e., the prefrontal cortex and anterior cingulate) and again found significant numbers of memory-selective neurons (Fig. 1). In the prefrontal cortex, we found 22 such neurons (6 repetition detectors and 16 novelty detectors) out of 276 recorded neurons (8.0%). In the anterior cingulate, we found 47 memory-selective neurons (18 repetition detectors and 29 novelty detectors) out of 344 recorded neurons (13.7%). Each count in these areas was higher than expected by chance (prefrontal cortex: 276 × 0.05 = 13.8, *P* < 0.05; anterior cingulate: 344 × 0.05 = 17.2, *P* < 0.001). Thus, the generic memory signal was found in every brain region that we examined.

### Identifying the Item-Specific, Sparsely Coded Memory Signal.

A sparsely coded signal can be identified by comparing the shapes of the full distributions of normalized spike counts for novel items vs. repeated items. If the episodic memory signal reflects sparse coding, then the repeated-item distribution should selectively exhibit a small percentage of outliers. These outliers would reflect the strong response of a small percentage of neurons to a small percentage of repeated items. If present, the outliers would be expected to increase the first four moments of the repeated-item distribution (mean, variance, skewness, and kurtosis) relative to the novel-item distribution, with skewness and kurtosis being much more sensitive than the mean and variance (25).

**Fig. 2.**
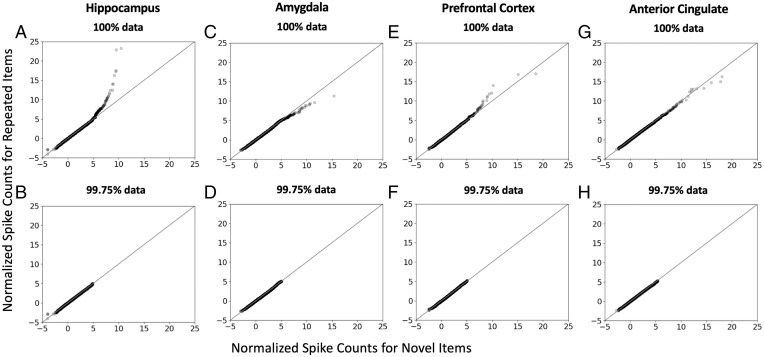
QQ plots of the normalized spike counts in response to novel words (*x*-axis) and repeated words (*y*-axis) for the hippocampus (*A* and *B*), amygdala (*C* and *D*), prefrontal cortex (*E* and *F*), and anterior cingulate (*G* and *H*). *Top* panels plot 100% of the data for each region (hippocampus: 77,431 recordings; amygdala: 65,219 recordings; prefrontal: 47,399 recordings; anterior cingulate: 62,205 recordings). The dark symbols indicate high-density data points, representing thousands of recordings; the light symbols indicate low-density data points, representing a very small percentage of recordings. *Bottom* panels show the same data after removing the 0.25% recordings with the highest spike counts from both the novel-word and repeated-word distributions. For the hippocampus, the points fell mostly along the diagonal line but exhibited a sharp upward deflection (*A*), indicating that some neurons responded strongly to some repeated words. After removing the top 0.25% of the data, the upward deflection for the hippocampus disappeared (*B*), indicating that only a very small percentage of neurons spiked more in response to repeated words than novel words. By contrast, the plots for the other three regions did not exhibit similar upward deflections. Thus, the activity pattern signaling episodic memory was identified only in the hippocampus.

A visual method for comparing the shapes of two distributions is to generate empirical quantile-quantile (QQ) plots (26). Quantiles refer to rank-ordered data, broken into subgroups containing equal percentages of the data. For our analyses, the relevant empirical QQ plots consist of the quantiles of normalized spike counts for novel words (*x*-axis) vs. the quantiles of normalized spike counts for repeated words (*y*-axis), constructed separately for each of the four brain regions (Fig. 2). If these two distributions of normalized spike counts have the same shape (e.g., two log-normal distributions), the QQ plot will be linear, even if the distributions have different means and/or SDs. However, if the repeated-item distribution is more skewed to the right than the novel-item distribution, as predicted by neurocomputational models, the QQ plot will instead exhibit a characteristic, nonlinear deflection.

For the hippocampus, the points on the QQ plot fall mostly along the diagonal line but show a sharp upward deflection for the highest-ranking points (Fig. 2*A*, see *SI Appendix* for representative raster plots). As indicated by the light symbol shading at the rightmost portion of the QQ plot, the deflection reflects a very small percentage of recordings that exhibited markedly strong responses to specific repeated words. Indeed, when only 0.25% of the top-ranking recordings from both distributions were removed, retaining 99.75% of the recordings, the upward deflection was no longer apparent (Fig. 2*B*). This is evidence of sparse coding in hippocampal neurons. By contrast, similar patterns of sharp upward deflections were not observed in the amygdala, prefrontal cortex, or anterior cingulate (Fig. 2 *C*–*H*).

We next tested whether the nonlinear QQ plot apparent visually in the hippocampus reflected a statistically reliable departure from linearity. We did so by testing whether the skewness and kurtosis for the repeated- and novel-item distributions differed significantly according to a bootstrap test (see *Materials and Methods*). Indeed, although both distributions were positively skewed (repeated: skewness = 2.77; novel: skewness = 2.09), the repeated distribution was significantly more skewed (*P* = 0.002). The repeated distribution also had significantly higher kurtosis than the novel distribution (20.11 vs. 6.44, *P* = 0.006). By contrast, in the other three brain regions, neither skewness nor kurtosis differed significantly across the repeated vs. novel distributions (Table 1), consistent with the interpretations from visual inspections of the QQ plots.

**Table 1.** Statistical moments of the distributions of normalized spike counts associated with single-unit recordings made to novel and repeated items

	Hippocampus		Amygdala		Anterior cingulate		Prefrontal	
	Repeated	Novel	Repeated	Novel	Repeated	Novel	Repeated	Novel
Mean	−0.007	−0.015	0.039	0.025	0.032	0.037	−0.023	−0.042
SD	0.99	1.007	1.049	1.022	1.053	1.041	0.978	0.988
Skewness	2.77	2.09	1.97	2.02	2.43	2.53	2.84	2.62
Kurtosis	20.11	6.44	5.75	6.69	11.49	12.84	14.43	11.91

With regard to both skewness and kurtosis, the difference between repeated vs. novel items was significant only in the hippocampus (*P* = 0.002 and *P* = 0.006, respectively). With regard to both the mean and the SD, the difference between the repeated vs. novel item spike-count distributions was not significant in any of the four brain regions. The mean and SD are less sensitive to the presence of outliers than are skewness and kurtosis (25).

Next, we tested the interaction between the skewness difference in the hippocampus vs. the skewness difference in the other three brain regions. The interaction between the hippocampus and the amygdala (*P* = 0.001) and the interaction between the hippocampus and the anterior cingulate cortex (*P* = 0.002) were both highly significant, whereas the interaction between the hippocampus and the prefrontal cortex just missed being significant (*P* = 0.057). For kurtosis, all three interactions were significant (*P* = 0.004 for the hippocampus vs. the amygdala; *P* = 0.007 for the hippocampus vs. the anterior cingulate; and *P* = 0.043 for the hippocampus vs. the prefrontal cortex).

We next asked whether the findings in the hippocampus might be based on only a few patients, sessions, neurons, or stimuli (Table 2). In fact, the top 0.25% of normalized spikes to repeated words came from 25 different patients (out of 30 patients with single-unit data from the hippocampus) across 38 different sessions (out of 51 sessions with single-unit data from the hippocampus). In addition, each of these 38 sessions contributed 1 to 11 unique single neurons to the top 0.25% (mean = 2.95 neurons per session). On average, each of these neurons responded strongly to 1.66 unique repeated words (range = 1–11).

**Table 2.** Characteristics of the recordings from the hippocampus

	100% data	0.25% data
Recordings (word presentations x recorded neurons)	77,431	186
Patients	30	25
Sessions	51	38
Unique neurons	396	112
Unique words	821	144
Average unique neurons per session	7.76	2.95
Average unique words per neuron	187.96	1.66
Average unique neurons per word	7.95	1.18

## Discussion

In this study, we found that individual hippocampal neurons exhibit two distinct memory signals. One signal is generic: the recorded neurons respond differentially depending on the categorical status of test items (novel vs. repeated). In each region, ∼10% of neurons were either novelty detectors or repetition detectors, responding consistently across trials. This signal was also widespread, as it was found in all four brain regions that we examined (hippocampus, amygdala, prefrontal cortex, and anterior cingulate). The other memory signal was item-specific and pattern-separated: each neuron responded strongly to a small fraction of repeated words (∼1.66 words), and each repeated word elicited strong responding in a small fraction of neurons (∼1.18 neurons). These neurons are different from concept neurons (discussed below), which fire strongly whenever the item is presented, both on the first presentation in the experimental context (i.e., when the item was novel) as well as on the second presentation (when the item was repeated). The item-specific episodic memory signal was found in (and only in) the hippocampus. The coexistence of these two memory signals (i.e., generic vs. item-specific) in the same set of neural recordings has not been previously documented, nor has the regional specificity of those signals (i.e., widespread vs. hippocampus-specific).

Neurocomputational models (18–21) predict that item-specific episodic memories are sparsely coded in the hippocampus and with largely nonoverlapping neural ensembles (pattern-separated). Applied to the continuous recognition task, these models predict that, for test items equated in every respect except their episodic occurrence in the experimental context (novel vs. repeated), these signals should be selectively detected for repeated items. To detect them, we compared the shapes of neuron-by-item normalized spiking distributions for novel vs. repeated items (22, 23). Unlike the standard method used to detect the generic memory signal, the method of analysis that detected the item-specific signal is not intuitive and to our knowledge has not been used by other laboratories investigating the neural representation of memory. To employ this method would presumably require an explicit intention to test the predictions of neurocomputational models (27). Our analyses showed that episodic memory is very sparsely coded (detected in only ∼0.25% of the data), item-specific, pattern-separated, and found only in the hippocampus. We therefore suggest that this signal represents the episodic memory of individual items/events and is fundamental for the formation of episodic long-term memory.

Previous single-unit studies with humans typically searched in the medial temporal lobe only for the generic memory signal, finding it in the amygdala and the hippocampus (15–17). We detected the generic memory signal not only in both the amygdala and the hippocampus but also in areas beyond the medial temporal lobe (i.e., in the prefrontal cortex and anterior cingulate cortex). Similarly, a recent study found the generic signal outside the medial temporal lobe, in parietal cortex (28). Therefore, this signal is not only generic but is also widespread in the brain.

What role might the widespread generic memory signal play? Hippocampal neurons that sparsely code item-specific, episodic memories may distribute generic information to other brain regions (and to other neurons in the hippocampus) to perform memory-related functions not requiring item-specific information. For example, the prefrontal cortex may determine whether the memory signal for a test item is strong enough to exceed a decision criterion. Because that decision would evaluate memory strength, as represented by the generic signal, item-specific content would not be required. Similarly, parietal cortex may play a role in assessing confidence (29, 30), which again requires only information about memory signal strength. On this view, the item-specific memory signal in the hippocampus is fundamental, coding episodic memory for individual items/events, whereas the generic memory signal—the focus of much prior research—is secondary and derivative.

The item-specific and generic memory signals reported here invite an interpretation in terms of recollection and familiarity, respectively (31). One suggestion has been that the hippocampus selectively supports recollection whereas a different structure, such as perirhinal cortex, supports the generic familiarity memory signal (32, 33). However, contemporary models of human recognition memory hold that the familiarity signal is determined by contextual associations (the word is familiar because it was encountered on an earlier list in the experimental context) even in the absence of the experience of recollection (). Moreover, the association between an item and the experimental context is widely thought to require the hippocampus (1, 21, 38). This would explain, for example, why patients with bilateral lesions limited to the hippocampus exhibit an impairment in familiarity in addition to an impairment in recollection (5, 39).

Our findings contradict a recent claim (40) that episodic memories in the human hippocampus are not stored as sparsely coded, pattern-separated representations. The claim instead is that concept neurons code episodic memories. Concept neurons fire selectively when specific concepts (e.g., “Eiffel Tower”) are evoked, whether the evoking stimulus is an image representing the concept, its printed name, or its spoken name (41). Critically, concept neurons can expand their tuning, responding to unrelated stimuli that have been paired with the relevant concept (42). For example, if the Eiffel Tower and the actor Jackie Chan are shown together during an experiment, the “Eiffel Tower neuron” will subsequently increase its firing rate to a Jackie Chan image presented alone.

In our view, these neural signals do not reflect episodic memories. Concept neurons respond to any stimulus that evokes their preferred concepts (e.g., Jackie Chan evoking the concept of the Eiffel Tower), suggesting that they code factual knowledge (i.e., semantic memory). Episodic memory (as in the present study) carries what, when, and where information about the test stimuli (3). As noted by others (43, 44), the tasks used in concept cell studies do not require patients to appreciate such specific information. Perhaps that is why concept neurons, like the widespread generic memory signal, have been found in brain areas besides the hippocampus (41). By contrast, our study required patients to appreciate precisely that kind of information, and it yielded clear evidence of sparsely coded, pattern-separated episodic memory signals in the hippocampus. Studies using other methodologies have also identified pattern-separated memory signals in the hippocampus (45–47).

In our experiment, concept neurons that respond to semantic meanings would have responded to both presentations of a word (novel and repeated). By contrast, we observed neurons that fired differentially for particular repeated words and therefore integrated stimulus information about the attributes of “what” (i.e., this particular word), “when” (presented a few minutes ago), and “where” (in this experimental context). This item-specific neural code for episodic memory was sparse, pattern-separated, and limited to the hippocampus.

## Materials and Methods

### Participants.

The participants were 34 patients (mean age, 41 ± 2.02 y; 21 females and 14 males; all but 2 were right-handed) with drug-resistant temporal lobe epilepsy that required implantation of depth electrodes (Ad-Tech Medical) for clinical evaluation and consideration of possible surgical resection of their seizure foci. The patients completed 55 total sessions, with individual patients completing 1 to 4 sessions. An additional 10 sessions from 6 of the 34 patients and all of the data from 1 additional patient were excluded from analysis because of low recognition memory performance (*d*′ no greater than 0).

### Informed Consent.

Decisions about whether to implant the depth electrodes and where to implant them were made by their treating neurologists and were based solely on clinical criteria. Patients who were deemed to require depth electrodes were provided with a copy of surgical consent forms. They were also made aware of the risks and benefits in undertaking depth electrode implantation and intracranial monitoring as well as the subsequent surgical resection.

Decisions pertaining to microwire placement and recording for research purposes were independent from the clinical decisions about implanting the depth electrodes. The potential placement of microwires was discussed with the patient and their family. After all concerns were discussed, the patients were asked if they would agree to microwire placement. If they did agree, they then signed a statement of consent to microwire placement and recording (see *SI Appendix* for additional information). All patients provided informed consent to participate in the research, using a protocol approved by the Institutional Review Board of St. Joseph’s Hospital and Medical Center.

### Microwire Implantation.

The extracellular potentials corresponding to single-neuron activity were recorded from the tips of 38-μm-diameter microwires implanted along with a clinical depth electrode (Ad-Tech Medical Corporation, Racine, WI) used to record clinical field potentials (48, 49). Each depth electrode contained a bundle of nine microwires, implanted stereotactically (Medtronic Stealth Station) using a 3 T structural MRI. Target locations were chosen by selecting a trajectory for the depth electrode which positioned the low-impedance contacts along the shaft of the depth electrode in clinically mandated areas with the tip of the depth electrode ∼3 mm superficial from the deepest part of a target structure, such as the hippocampus. Electrodes were inserted through a skull bolt with a custom frame to align the depth electrode along the chosen trajectory. The error in tip placement using this technique is estimated to be ±2 mm, based on manual inspection of preoperative MRI and postoperative computed tomography in several cases, and is similar to that previously reported) for a laterotemporal approach (50). While this accuracy is insufficient to determine subfields within the hippocampus or nuclei within the amygdala, it does ensure that the microwires are within the target brain area.

### Experimental Design.

The patients were tested using continuous recognition memory: words were presented in a series, with most words repeated after 0, 1, 3, 7, 15, or 31 intervening words. The task was to judge whether each word was novel (i.e., presented the first time) or repeated (i.e., presented a second time). The words were presented either visually on a computer screen or auditorily through headphones. Across 55 sessions (from 34 patients), there were 35 visual sessions and 20 auditory sessions. QQ plot data from the visual sessions (focusing on recordings from the hippocampus and amygdala only) were analyzed in a previous report (22). Single-unit recordings from each structure were available for most of these sessions (hippocampus = 51 sessions, amygdala = 45 sessions, prefrontal cortex = 38 sessions, and anterior cingulate = 45 sessions). As is typically done, we treated these 55 sessions as if they were independent, although some patients completed more than one session. Different sessions for any given patient were conducted on different days; it is typically assumed that different neurons were recorded during each session, as depth electrodes shift slightly as patients move around.

For the visual sessions, 360 words (120 one-syllable, 120 two-syllable, and 120 three-syllable words) were used, each of which was repeated once. The words were taken from the Medical Research Council Psycholinguistic database (51). Another set of 45 one-syllable words were used as fillers that were never repeated. Three nonoverlapping word sets could be presented; these were used for patients who volunteered for multiple sessions. Each session comprised 255 trials (240 trials wherein each of 120 words were presented twice along with15 filler trials). One patient completed more than four sessions, and thus saw one repeated set of words, but this repetition did not affect memory performance. In each trial, a word was visually displayed on a computer screen for 1,500 ms, followed by a question mark as a response prompt. Patients had up to 2,000 ms to indicate whether the word was novel or repeated. Trials ended when responses were entered. There was a jittered intertrial interval (mean = 888 ms; SD = 552 ms). (In some trials of the visual task, the prestimulus time period included the time when patients made their response on the previous trial. Excluding those trials from the analysis had a negligible effect on the results.) The procedure for the auditory sessions was similar: Each auditory session included 615 trials wherein 300 unique words were repeated once and 15 filler words were presented once. The response prompt appeared at the end of the audio file for the trial. There was a jittered intertrial interval that lasted for an average of 1,055 ms with a SD of 53 ms. Data are available at the Open Science Foundation repository at https://osf.io/9tgmx/. This is the same data set that was analyzed by Urgolites et al. (52) to investigate a different issue, namely, the effect of prestimulus activity on subsequent memory.

### Statistical Analysis.

We first identified neurons that responded differently to words that were novel vs. repeated. For each neuron in each brain region, raw poststimulus spike counts from 200-1,000 ms after presentation of the repeated or novel item (i.e., from 200-1,000 ms after presentation of the visual words for the visual sessions and from 200-1,000 ms after completion of the word sound file for the auditory sessions) were compared for novel and repeated words, using paired *t* tests. Neurons were classified as “repetition detectors” if they spiked more to repeated words than novel words (*P* < 0.05), and as “novelty detectors” if they spiked more to novel words than repeated words (*P* < 0.05). Raw spike counts were used, as in earlier studies that identified repetition and novelty neurons (15–17).

Second, we assessed the sparse coding signal in all four brain regions. For every recorded neuron, we computed poststimulus (200-1,000 ms after stimulus presentation) normalized spike counts for each trial (*i*), where a “trial” refers to a word presentation. For each neuron (*j*), baseline spike counts (1,000-200 ms before the onset of the word) were computed across all trials in that session (mean and SD of spike counts, μ*_j_* and σ*_j_*, respectively). Normalized poststimulus spike counts for a given trial (*N_ij_*) in which *s_ij_* raw spike counts were recorded on trial *i* for neuron *j* is given by *N_ij_* = (*s_ij_* − μ*_j_*)/σ*_j_*. A quantile-quantile (QQ) plot was generated for each brain area for the full distribution of rank-ordered, normalized spike counts for novel and repeated items. Each distribution contained the spike count for all recorded neurons in response to all test stimuli across all patients and sessions. Skewness and kurtosis were also calculated for each distribution. We conducted bootstrap analyses to examine whether the skewness and kurtosis differed reliably for the novel- and repeated-item distributions. For each test (e.g., comparing skewness for novel and repeated items in the hippocampus), 10,000 bootstrap trials were performed. In each case 1) the normalized poststimulus spike counts from all repeated words (*n_Repeated_*) and all novel words (*n_Novel_*) were combined; 2) *n* spike counts were randomly sampled with replacement from the combined dataset and labeled as *n_Repeated_* bootstrap “targets,” and then *n* spike counts were randomly sampled with replacement from the combined dataset and labeled as *n_Novel_* bootstrap “foils”; and 3) the difference between the statistic of interest (e.g., skewness) of those two bootstrap samples was computed. The proportion of the 10,000 bootstrap trials in which the absolute value of the difference was greater than the observed difference yielded the *P* value.

A similar bootstrap method was used in analyzing the interaction of the difference in a statistic (e.g., skewness) for novel and repeated items between the hippocampus and each of the other three brain regions. For each analysis, 10,000 bootstrap differences between novel and repeated items in the hippocampus were compared with 10,000 bootstrap differences between novel and repeated items for another region (e.g., amygdala). This generated 10,000 difference scores between the difference scores from within each of two regions (i.e., a difference between two sets of difference scores). The proportion of the 10,000 trials that had a difference greater than the observed difference between the two regions yielded the *P* value.

## Supplementary Material

Supplementary File

## Data Availability

Anonymized single-neuron data have been deposited in Open Science Foundation (https://osf.io/9tgmx/) (53).

## Footnotes

*The term “familiarity detector” has been used by others to refer to neurons that respond more to repeated items than to novel items. However, the term “familiarity” is often used as well to refer to a theoretical memory signal that is neither context-dependent nor hippocampus-dependent. We therefore use the more theoretically neutral term “repetition detector.”

## Change History

May 6, 2022: Due to a production error, Tables 1 and 2 appeared incorrectly. The tables have been updated.
