# Supplementary material for: Two kinds of memory signals in neurons of the human hippocampus
Source: Proc Natl Acad Sci U S A. 2022 May 5;119(19):e2115128119. doi: 10.1073/pnas.2115128119 (PMC9171769; doi:10.1073/pnas.2115128119)
Supplement: Supplementary File [file pnas.2115128119.sapp.pdf]

Supporting Information

Microwire Implantation. The extracellular action potentials corresponding to single-neuron activity and local field potentials were recorded from the tips of microwires implanted bilaterally along with a clinical depth electrode used to record clinical field potentials (1, 2). The implantation sites were chosen according to clinical criteria, which limits the potential recording sites. For the patients studied here, however, the sites included the hippocampus, prefrontal cortex, anterior cingulate cortex, and amygdala, all bilaterally. In the hippocampus, the wires were targeted to be in the midbody of the hippocampus, just behind the head of the hippocampus, opposite the apex of the cerebral peduncle. In the prefrontal cortex, the wires were targeted to be in the ventromedial prefrontal cortex, below the anterior cingulate gyrus. In the anterior cingulate, the wires were targeted to be in the parts above and behind the genu of the corpus callosum. In the amygdala, the wires were targeted to be in the center of that structure.

Each recording site received a bundle of nine 38 μm diameter platinum-iridium microwires (California Fine Wire), implanted stereotactically (Medtronic StealthStation) using a 1.5 T structural MRI. Electrodes were placed through a skull bolt with a custom frame to align the depth electrode along the chosen trajectory. The error in tip placement using this technique is estimated to be ± 3 mm based on manual inspection of the pre-operative MRI and post-operative CT and prior work (3, 4).

Pre-onset spike counts were recorded 200 - 1000 ms before the onset of the test stimulus for both the visual and auditory sessions. Post-onset spike counts for each cluster were recorded 200 -1000 ms after the onset of the visual stimulus and 200 - 1000 ms after the offset of the sound file for the auditory stimuli. The test period during which post-stimulus spike counts were recorded was chosen because a previous study (5) found that selective responses of hippocampal

neurons began ~300 ms after stimulus onset and because nearly all behavioral responses occurred after 1 s.

Electrodes and Microwires Decisions. In the protocol used at the Barrow Neurological Institute, decisions about whether to implant the depth electrodes and where to implant them are based solely on clinical criteria and are completely independent of decisions pertaining to microwire placement and recording.

Patients were selected for depth electrode implantation at the Barrow Neurological Institute following the protocol approved by the St. Joseph's Hospital Institutional Review Board. Patients are selected based on well-defined clinical criteria in line with generally recognized diagnostic practices (6, 7). The decision to offer intracranial monitoring of seizures to a patient and the planning of the clinical study is made by the treating epileptologist with advice from the participants in the weekly case conference. Only those patients for whom noninvasive tests fail to narrow down the seizure focus sufficiently for adequate planning of the surgical resection have depth electrodes. Patients are told of the decision by their treating neurologists. They are then seen by their neurosurgeon. At this time, they are provided with a copy of the surgical consent forms and made aware of the risks and benefits in undertaking depth electrode implantation and intracranial monitoring as well as the subsequent surgical resection.

Thus, decisions about placement of the depth electrodes involve many physicians other than the potential clinical co-authors on these papers, Drs. Smith (the neurosurgeon) and Treiman, and are independent of any research concerns. No patient was ever referred for depth electrode placement by Dr. Treiman without the consensus of the EMU conference participants.

The placement of the depth electrodes in these four brain areas, including the anterior cingulate cortex and ventromedial prefrontal cortex, was a longstanding practice adopted by the clinical team in ~1995. These sites were implanted to check whether the patient's seizures had an extratemporal origin which would then change recommendations for further surgery. The patients in this study had their depth electrodes implanted and participated in this research between 2006 and 2014. While practices for epilepsy monitoring vary by center and have changed over time, in this 2006-2014 timeframe, the routine practice of the clinical team at the BNI was to implant electrodes bilaterally in four brain areas, the amygdala, hippocampus, anterior cingulate cortex, and ventromedial prefrontal cortex. As noted above, these areas were chosen based solely on clinical criteria.

The addition of microwires to the clinically required depth electrodes for research purposes was approved by the Institutional Review Board of St. Joseph's Hospital and Medical Center. The protocol for the addition of microwires was originally submitted in August 2005 and was approved after 2 months of review in October 2005. The protocol described the exact steps taken for preparation and placement of the microwires and included a diagram of the relative sizes of the depth electrode and microwires. A separate section described the long history of safe use of this type of combination of depth electrodes and microwires at UCLA, where Dr. Steinmetz trained.

The following is a photograph showing the scale of the clinically required depth electrode on the left with the microwires protruding to the right. The depth electrode is 1.8 mm in diameter and is typically inserted to a depth of 8-10 cm laterally though the temporal cortex. There are 9

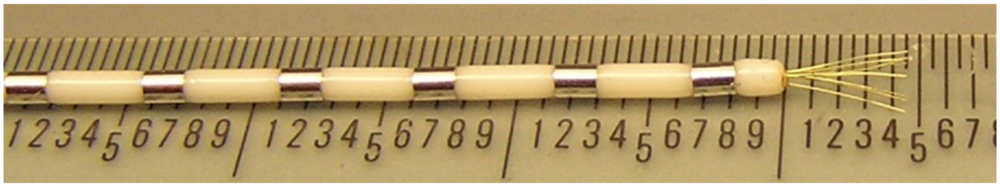

microwires in the bundle, each of which is 38 μm in diameter and they protrude 5 mm from the tip of the depth electrode. The microwires thus represent little additional penetration of the brain tissue beyond what is clinically required.

Patients were provided a detailed written description of the nature of the microwires and the purpose of recording after meeting with the neurosurgeon to discuss the need for the depth electrode recordings and the surgery to implant the depth electrodes. They were asked to consider whether they would like to participate in the time between this meeting and their next meeting prior to surgery.

At that next meeting a member of the research team, typically Dr. Steinmetz, discussed the protocol with the patient and their family and addressed any questions they might have had about the microelectrodes, their placement, and the type of experimental tasks they would be asked to perform while in the epilepsy monitoring unit. At this meeting, which usually ranged between 15 to 30 minutes, the patients were explicitly asked if they would like to see an example of the electrodes and microwires. In most cases, the patients did wish to see and touch them. Only after all concerns were discussed were the patients asked if they would agree to microwire placement. If they did agree, they then signed the written statement of consent to microwire placement and recording.

In a recent article noting that it now seems appropriate to use microwire recordings to clinically benefit the patients, Chari, Thornton, Tisdall and Scott (8) note that "*In our systematic search (see Supplementary material), none of the studies examining electrode design and implantation safety reported increased complication rates as a result of combining research microelectrode recording with clinical macroelectrodes, illustrating the safety of these techniques.*" The same techniques are presently used in at least 6 centers around the world. In all

of these, the use of the microwires and recording for research purposes has been reviewed and approved by the appropriate institutional review boards.

In all, we always ensured that the placement of microwires and subsequent recording presented little additional risk to them. We also ensured that the patients fully understood the nature of the recordings and microwires and were completely free to make their own decision on whether or not to participate based upon their desire to participate in such scientific research.

Filtering and Event Detection. Extracellular potentials were recorded from the tips of the microwires using techniques previously described (9) and digitized at 29,412 Hz with 16-bit resolution. Possible action potential events (APs) were detected using digital filtering and thresholding (10). Because more than one neuron may be recorded near any given electrode, APs were sorted into several clusters of similar waveform shape using the open-source clustering program KlustaKwik (Klustakwik.sf.net). After sorting, each cluster was graded as being noise, multiunit activity (MUA), or single-unit activity (SUA) based on criteria such as the waveform shape, size of the waveform relative to noise, evidence of a refractory interval, and lack of powerline interference, using the criteria described previously (9).

In our experience, this technique produces results comparable to prior reports in other laboratories (10) in terms of recorded waveform shapes, interspike intervals, and firing rates. While it is important to note that these and other reports of human single-unit recordings (11) do not achieve the quality of unit separation achievable in animal recordings (12) they nonetheless represent neural activity at a much finer spatial and temporal scale than is achievable using other methods such as fMRI.

Behavioral Performance at different lags. Each of the 6 lags (i.e., with 0, 1, 3, 7, 15, or 31 intervening words) had an equal number of trials for both the visual and auditory sessions. For the visual sessions, 120 unique words were repeated, and 20 words were repeated at each lag. For the auditory sessions, 300 unique words were repeated, and 50 words were repeated at each lag. As is typical, recognition performance was better at shorter lags. At the six lags, patients were 87.5%, 81.6%, 81.0%, 75.9%, 72.5%, and 70.3% correct in recognizing a previously presented word, respectively. There was no ceiling effect at 0 or 1 lag. This is probably because it was a continuous recognition task in which, for each trial, participants needed to hold as many previous words as possible in their working memory or else they relied on long-term memory. This is more complicated than a pure working memory task, wherein the participant only needs to maintain one stimulus per trial.

Supporting Figures

1. Raster plots of raw spikes for two novelty detectors of the generic memory signal.

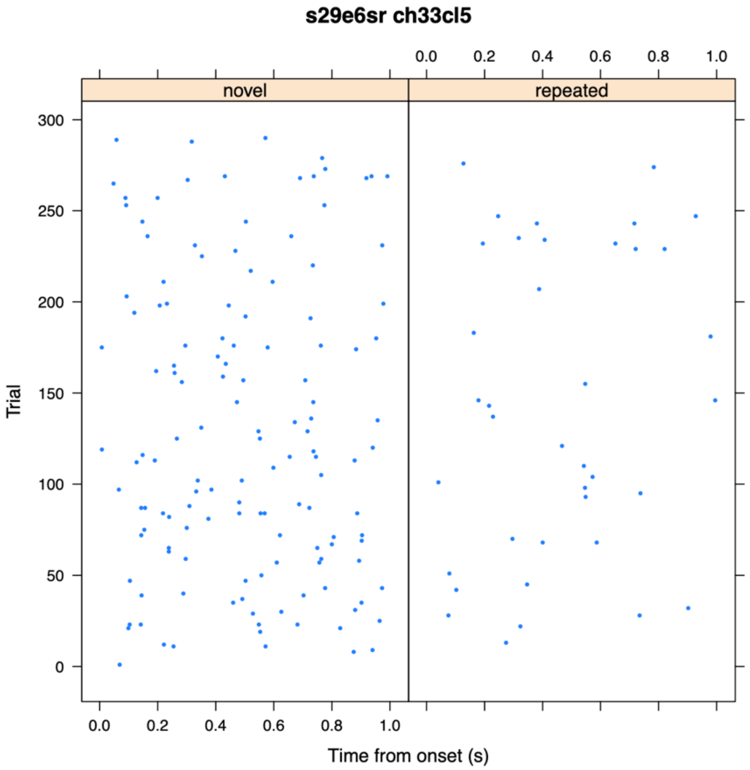

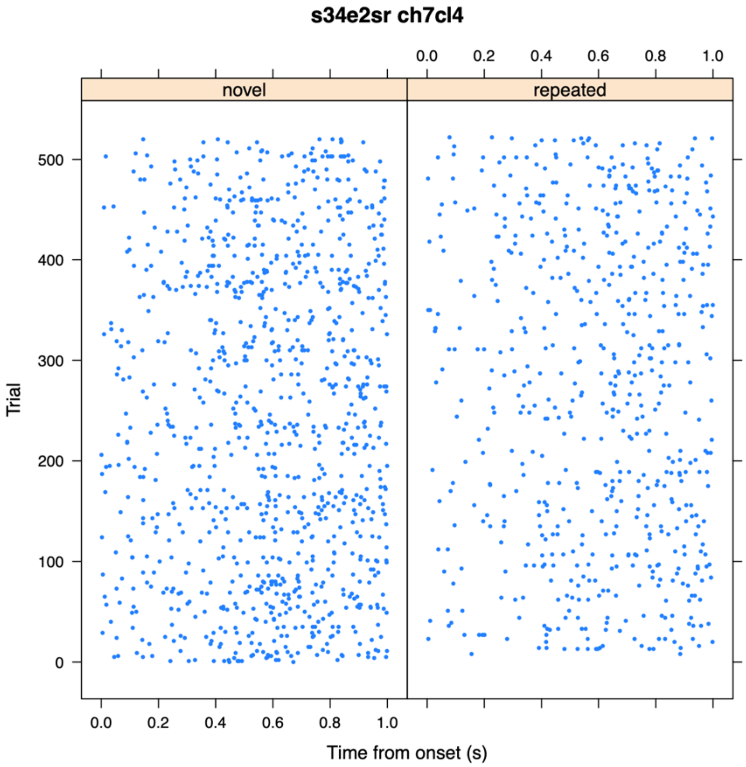

2. Raster plots of raw spikes for two repetition detectors of the generic memory signal.

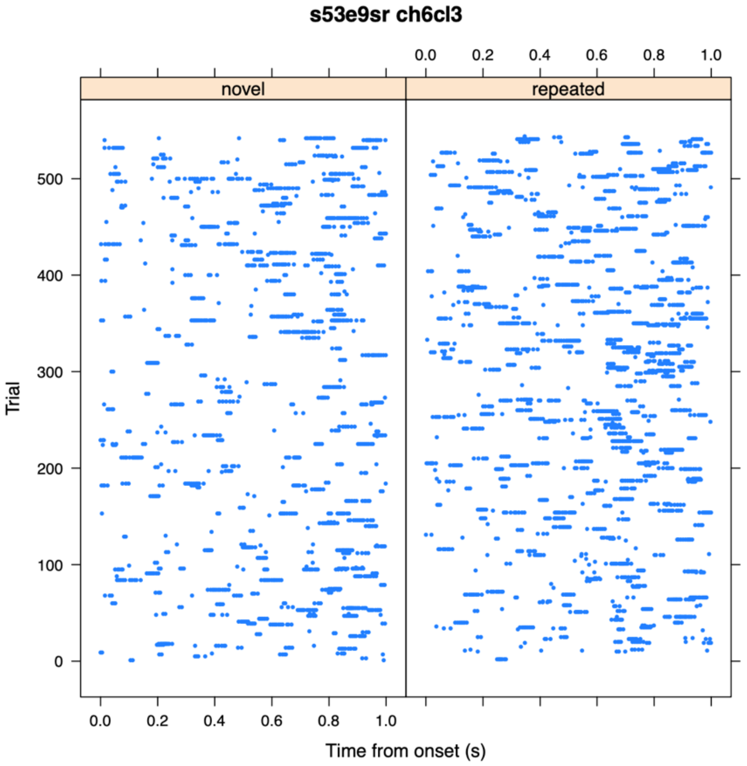

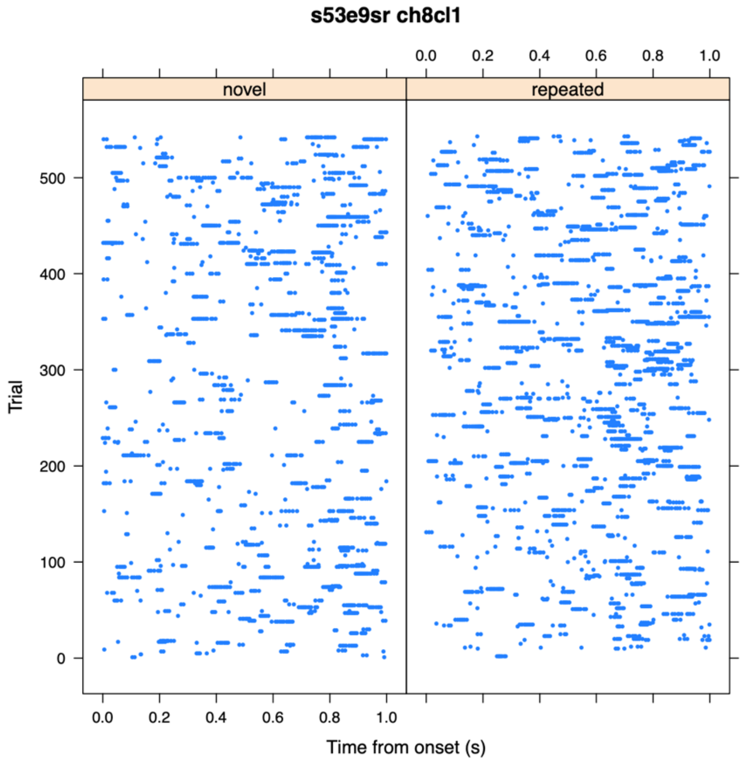

3. Two raster plots of raw spikes for the item-specific memory signal.

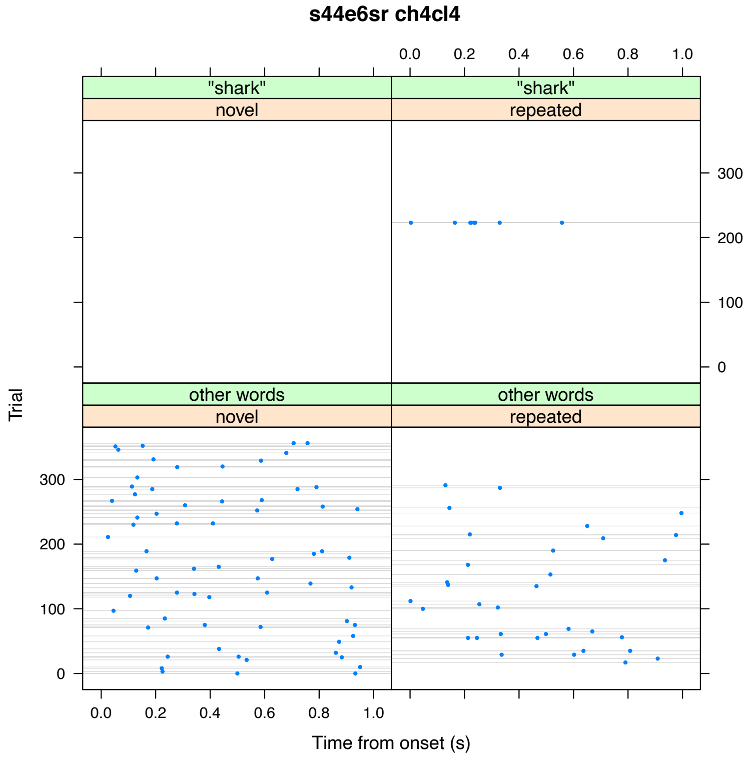

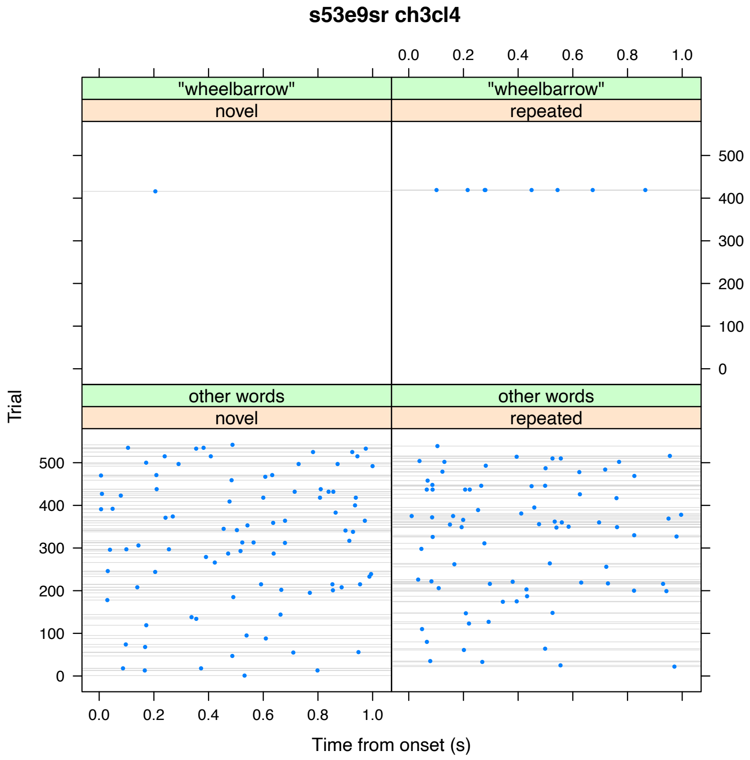

References

1. A. M. Dymond, T. L. Babb, L. E. Kaechele, P. H. Crandall, Design considerations for the use of fine and ultrafine depth brain electrodes in man. *Biomed Sci Instrum* **9**, 1-5 (1972).
2. I. Fried *et al.*, Cerebral microdialysis combined with single-neuron and electroencephalographic recording in neurosurgical patients. Technical note. *J Neurosurg* **91**, 697-705 (1999).
3. M. Nowell *et al.*, A novel method for implementation of frameless StereoEEG in epilepsy surgery. *Neurosurgery* **10 Suppl 4**, 525-533; discussion 533-524 (2014).
4. K. Roessler *et al.*, A Frameless Stereotactic Implantation Technique for Depth Electrodes in Refractory Epilepsy Using Intraoperative Magnetic Resonance Imaging. *World Neurosurg* **94**, 206-210 (2016).
5. R. Q. Quiroga, L. Reddy, G. Kreiman, C. Koch, I. Fried, Invariant visual representation by single neurons in the human brain. *Nature* **435**, 1102-1107 (2005).
6. J. Engel, Jr., P. H. Crandall, Intensive neurodiagnostic monitoring with intracranial electrodes. *Adv Neurol* **46**, 85-106 (1987).
7. J. Engel, Jr., M. F. Levesque, W. D. Shields, Surgical treatment of the epilepsies: presurgical evaluation. *Clin Neurosurg* **38**, 514-534 (1992).
8. A. Chari, R. C. Thornton, M. M. Tisdall, R. C. Scott, Microelectrode recordings in human epilepsy: a case for clinical translation. *Brain Commun* **2**, fcaa082 (2020).

9. A. B. Valdez, E. N. Hickman, D. M. Treiman, K. A. Smith, P. N. Steinmetz, A statistical method for predicting seizure onset zones from human single-neuron recordings. *J Neural Eng* **10**, 016001 (2013).

10. I. V. Viskontas, B. J. Knowlton, P. N. Steinmetz, I. Fried, Differences in mnemonic processing by neurons in the human hippocampus and parahippocampal regions. *J Cogn Neurosci* **18**, 1654-1662 (2006).

11. G. Kreiman, C. Koch, I. Fried, Category-specific visual responses of single neurons in the human medial temporal lobe. *Nat Neurosci* **3**, 946-953 (2000).

12. D. N. Hill, S. B. Mehta, D. Kleinfeld, Quality metrics to accompany spike sorting of extracellular signals. *J Neurosci* **31**, 8699-8705 (2011).
